# SARS-CoV-2 Spillback to Wild Coatis in Sylvatic–Urban Hotspot, Brazil

**DOI:** 10.3201/eid2903.221339

**Published:** 2023-03

**Authors:** Ana Gabriella Stoffella-Dutra, Bruna Hermine de Campos, Pedro Henrique Bastos e Silva, Karolina Lopes Dias, Iago José da Silva Domingos, Nadja Simbera Hemetrio, Joilson Xavier, Felipe Iani, Vagner Fonseca, Marta Giovanetti, Leonardo Camilo de Oliveira, Mauro Martins Teixeira, Zelia Ines Portela Lobato, Helena Lage Ferreira, Clarice Weis Arns, Edison Durigon, Betânia Paiva Drumond, Luiz Carlos Junior Alcantara, Marcelo Pires Nogueira de Carvalho, Giliane de Souza Trindade

**Affiliations:** Universidade Federal de Minas Gerais, Belo Horizonte, Brazil (A.G. Stoffella-Dutra, B.H. de Campos, P.H.B. e Silva, K.L. Dias, I.J.S. Domingos, J. Xavier, L.C. de Oliveira, M.M. Teixeira, Z.I.P. Lobato, B.P. Drumond, M.P.N. de Carvalho, G. de Souza Trindade);; Fundação de Parques Municipais e Zoobotânica de Belo Horizonte, Belo Horizonte (N.S. Hemetrio);; Fundação Ezequiel Dias, Belo Horizonte (F. Iani); Organização Pan-Americana da Saúde, Brasília, Brazil (V. Fonseca);; University of Campus Bio-Medico di Roma, Rome, Italy (M. Giovanetti);; Fundação Oswaldo Cruz, Rio de Janeiro, Brazil (M. Giovanetti, L.C.J. Alcantara);; Universidade de São Paulo, São Paulo, Brazil (H.L. Ferreira, E. Durigon);; Universidade de Campinas, Campinas, Brazil (C.W. Arns)

**Keywords:** COVID-19, respiratory infections, severe acute respiratory syndrome coronavirus 2, SARS-CoV-2, SARS, coronavirus disease, zoonoses, viruses, coronavirus, spillback, natural infections, coatis, wildlife, urban parks, Brazil

## Abstract

We tested coatis (*Nasua nasua*) living in an urban park near a densely populated area of Brazil and found natural SARS-CoV-2 Zeta variant infections by using quantitative reverse transcription PCR, genomic sequencing, and serologic surveillance. We recommend a One Health strategy to improve surveillance of and response to COVID-19.

By November 2022, the COVID-19 pandemic had resulted in >630 million cases of disease worldwide (*1*). During the outbreak, natural occurrence of SARS-CoV-2 infections in animals was a hallmark; infections have been reported mainly in companion, domestic, captive, and farmed animals but also in wildlife (*2*,*3*). As of September 2022, the World Organisation for Animal Health had recorded 26 animal species infected with SARS-CoV-2 in 36 countries (*2*), indicating that the virus is able to cross the species barrier, thereby increasing risk of new transmission cycles and animal reservoirs (*2*,*3*). 

Coatis (*Nasua nasua*) from South America are small diurnal mammals (family Procyonidae) that are omnivorous, terrestrial, synanthropic, and opportunistic. Coatis interact easily with humans and are often seen foraging for human food, especially from trash (*4*,*5*). We investigated the transmission of SARS-CoV-2 to a coati population living in an urban park near a large anthropized area of Brazil.

We collected serum samples and anal and oral swab samples during February–August 2021 from 40 free-living coatis inhabiting Mangabeiras Municipal Park in Belo Horizonte, Brazil (Appendix Table, Figure 1). Trained professionals captured coatis during 4 periods (February, June, July, and August), using appropriate personal protective equipment (laboratory coats, gloves, N95 face masks, and face shields) in accordance with all biosafety guidelines. Ethics approval was obtained for this study (Appendix). 

Coatis were captured in Tomahawk Live Traps (https://www.livetrap.com/index.php) (70 cm × 35 cm × 40 cm) baited with banana pieces. Animals were anesthetized with Zoletil 100 (Virbac, https://vet-uk.virbac.com) by intramuscular injection (7–10 mg/kg body weight), clinically evaluated, identified, and marked with polypropylene earrings and microchips. After anesthesia recovery, each coati was released at their capture site.

We stored anal and oral swab specimens by using RNAlater (ThermoFisher Scientific, https://www.thermofisher.com) and extracted RNA by using QIAmp Viral RNA Mini Kits (QIAGEN, https://www/qiagen.com). We performed quantitative reverse transcription PCR targeting the nucleocapsid N1 and N2 regions (*6*) and sequenced PCR positive samples by using nanopore technology. We performed phylogenetic analysis by using IQ‐TREE2 (*7*) and maximum-likelihood reconstruction. 

We detected SARS-CoV-2 RNA in 2 (5%) female coatis that had no clinical signs of infection (Table). We obtained a complete genomic sequence from the anal swab specimen from coati 535 (99% average coverage). The genomic sequence of SARS-CoV-2 obtained from the anal swab specimen from coati 535 indicated this variant belonged to the Zeta lineage (B.1.1.28.2, P.2) (Figure). The P.2 variant was initially detected in the state of Rio de Janeiro, Brazil, in July 2020 and was considered a variant of interest (*9*).

**Table:** Specimens from 2 SARS-CoV-2 RNA–positive coatis in study of SARS-CoV-2 spillback to wild coatis in sylvatic–urban hotspot, Brazil*

Coati ID	Collection date	Sex	Sample	SARS-CoV-2†	N1 Count‡	N2 Count‡
C341	2021 Feb 17	F	Oral swab	Positive	33	37
			Anal swab	Negative	NA	NA
			Serum	Negative	NA	NA
C535	2021 Feb 18	F	Oral swab	Positive	20	24
			Anal swab	Positive	30	33
			Serum	Negative	NA	NA

*Oral and anal swab and serum samples were collected from 40 wild coatis inhabiting Mangabeiras Municipal Park in Belo Horizonte, Brazil. We performed quantitative reverse transcription PCR targeting the nucleocapsid N1 and N2 regions of SARS-CoV-2 RNA for each sample. ID, identification, NA, not applicable. †Specimens positive or negative for SARS-CoV-2 RNA by PCR. ‡PCR cycle threshold count.

**Figure:**
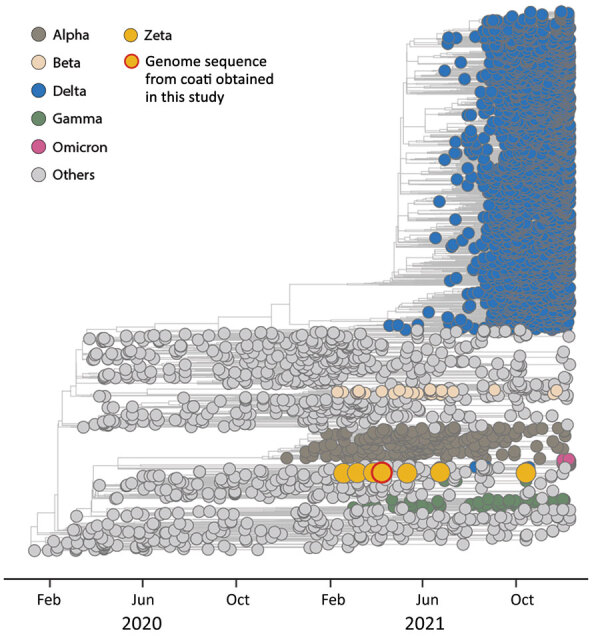
Time-scaled phylogenetic analysis of SARS-CoV-2 sequences, by variant type, in study of SARS-CoV-2 spillback to wild coatis in sylvatic–urban hotspot, Brazil. Maximum-likelihood method was used to compare the complete genomic sequence of SARS-CoV-2 obtained from coati (*Nasua nasua*) 535 (red-outlined yellow circle) and 3,441 SARS-CoV-2 reference genomic sequences (GISAID, https://www.gisaid.org) from around the world collected through October 2021. Colors represent clades corresponding to different SARS-CoV-2 variants of concern described by the World Health Organization; yellow indicates Zeta variant sequences. The SARS-CoV-2 sequence generated in this study was deposited in the GISAID database (accession no. EPI_ISL_8800460) and SisGen (Sistema Nacional de Gestão do Patrimônio Genético, https://www.sisgen.gov.br; no. A627307).

We performed plaque reduction neutralization tests (PRNT) on serum samples from all captured coatis to detect SARS-CoV-2 neutralizing antibodies (*8*). We serially diluted serum samples to obtain 1:20, 1:40, and 1:80 dilutions and measured 50% and 90% neutralizing activity against SARS-CoV-2. Twenty (50%) coatis had SARS-CoV-2 neutralizing antibodies in >1 dilution at the 50% level; at the 90% level, 13 (32.5%) coatis had detectable neutralizing antibodies in >1 dilutions and 7 (17.5%) coatis had SARS-CoV-2 neutralizing antibodies in all 3 dilutions. We observed neutralizing antibodies in all 3 serum dilutions for coati 535 (Appendix Figure 2).

We were unable to confirm the mode of SARS-CoV-2 transmission to the coati population. However, we found evidence for human-to-animal transmission; the P.2 genomic sequence from coati 535 was the same variant circulating in humans within the area during the study period. Furthermore, 50% of the coati population had antibodies against SARS-CoV-2, suggesting a cluster of natural exposure and infections within this population. Our results support indirect contact of coatis with contaminated human trash and food scraps in dumpsters and in the bordering urban areas of the park or potential direct close contact with infected human visitors (Appendix Figure 1).

Our findings agree with results from a zoo in Illinois, USA, that also confirmed SARS-CoV-2 in a coati by using molecular methods (*2*,*10*). Those results reinforce the susceptibility of coatis to SARS-CoV-2 infection and suggest possible virus shedding and transmission capacity of coatis. Viral RNA detection in both oral and anal swab specimens from coati 535 (Table) and presence of neutralizing antibodies indicate that viral replication occurred in this host. Therefore, our findings highlight possible SARS-CoV-2 enzootic maintenance in nature, including in fragmented green areas close to urban settings. Because of the potential for SARS-CoV-2 interspecies transmission, we recommend establishing a One Health strategy to improve surveillance and ability to respond to COVID-19 emergency health events.

Appendix Additional information for SARS-CoV-2 spillback to wild coatis in sylvatic–urban hotspot, Brazil.
