# Supplementary material for: SARS-CoV-2 Spillback to Wild Coatis in Sylvatic–Urban Hotspot, Brazil
Source: Emerg Infect Dis. 2023 Mar;29(3):664–7. doi: 10.3201/eid2903.221339 (PMC9973689; doi:10.3201/eid2903.221339)
Supplement: Appendix — Additional information for SARS-CoV-2 spillback to wild coatis in sylvatic–urban hotspot, Brazil. [file 22-1339-Techapp-s1.pdf]

Article DOI: https://doi.org/10.3201/eid2903.221339
EID cannot ensure accessibility for supplementary materials supplied by authors. Readers who have difficulty accessing supplementary content should contact the authors for assistance.

SARS-CoV-2 Spillback to Wild Coatis in Sylvatic–Urban Hotspot, Brazil

Appendix

Ethics Approval

The study was approved by SISBIO (Sistema de Autorização e informação em Biodiversidade, license no. 75831), Ethical Committee on Animal Use (CEUA) of Universidade Federal de Minas Gerais (protocol nos. 158/2020 and 100/2021), SisGen (Sistema Nacional de Gestao do Patrimonio Genetico, no. A627307), and Fundação de Parques Municipais e Zoobotânica de Belo Horizonte (protocol no. FU 004–2020).

Appendix Table. Numbers of captured coatis categorized by collection date, sex, and age in study of SARS-CoV-2 spillback to wild coatis in sylvatic–urban hotspot, Brazil*

	Sex		Age†			
Capture month	M	F	Young	Immature adult	Adult	Total
February	3	5	3	3	2	8
June	4	6	3	3	4	10
July	6	9	3	1	11	15
August	4	7	1	2	8	11
Total no. animals	17	27	10	9	25	44

*Collection comprised 40 coatis (*Nasua nasua*) (62.5% female and 37.5% male) and 4 additional recaptures. Live traps (n = 25) were placed at 5 strategic points of passage for coatis (5 traps set per point) in Mangabeiras Municipal Park in Belo Horizonte, Brazil. The 5 capture points were: Caraça (n = 19 captured coatis), Casa de bonecas (n = 6), Mirante (n = 3), Praça das águas (n = 6), and Trilha 12 (n = 10).
†Biometrics analyses were performed to estimate age.

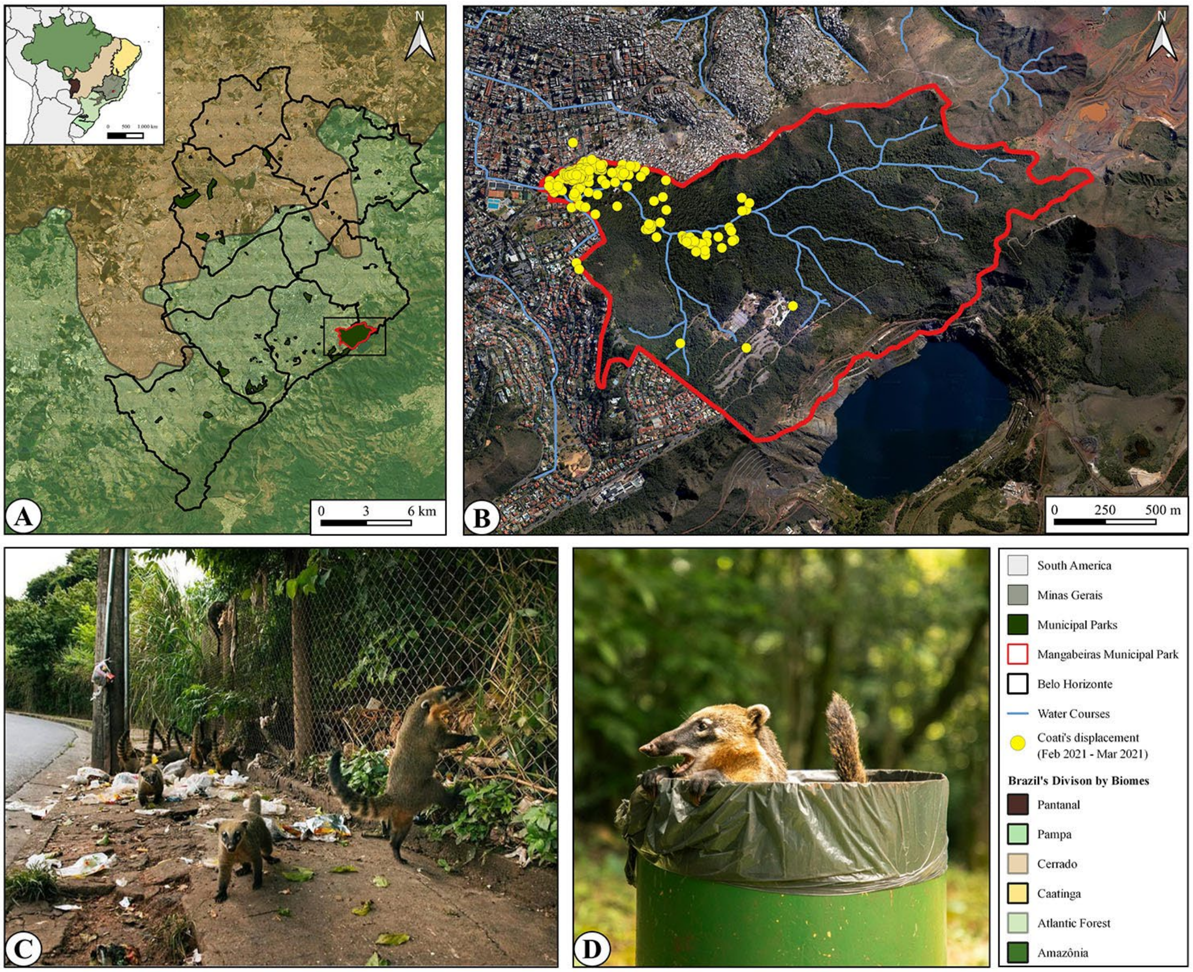

Appendix Figure 1. Overview of Mangabeiras Park in southeastern Brazil in study of SARS-CoV-2 spillback to wild coatis in sylvatic–urban hotspot, Brazil. A) Map of Brazil subdivided by its biomes and neighboring countries of South America, and municipal parks (dark green). Mangabeiras Municipal Park (19°56′ S and 43°54′ W) is surrounded by a red line. B) A satellite view of Mangabeiras Municipal Park (encircled by red line) in the city of Belo Horizonte highlighting the water courses (blue lines) and 341 coati displacements by georeferenced spots (global positioning system) (yellow circles) during February and March of 2021. Animal monitoring showed displacement throughout the park border areas that had residences and urban settings. C, D) Coatis in Mangabeiras Park in contact with human trash and food remnants. Photographer: Augusto Gomes. Maps were created with QGIS 3.24.3 software (https://www.qgis.org/pt_BR/site), using the coordinate reference system DATUM SIRGAS 2000 (UTM 23S).

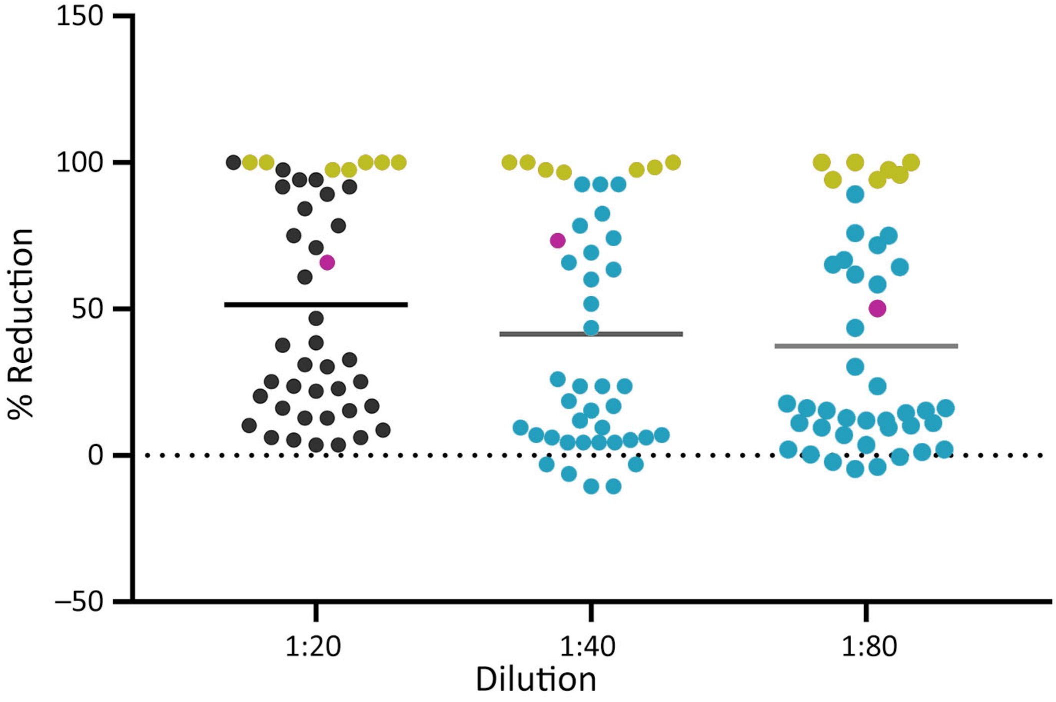

Appendix Figure 2. Scatter plot of plaque reduction neutralization tests of serum samples from coatis. SARS-CoV-2 neutralizing antibodies were measured in serum samples by using the plaque reduction neutralization test. Samples were serially diluted to 1:20, 1:40, and 1:80. Solid lines represent the average reduction percentage of all tested samples and dotted line is 0% reduction. Solid circles represent all samples tested at each dilution (n = 44) (4 additional samples were from recaptures; 3 of those showed seroconversion when compared with the previous collection date). Magenta circles represent coati 535, which was positive for SARS-CoV-2 RNA by PCR. Green circles represent 7 coatis that had 90% plaque reduction (neutralizing activity) in all 3 dilutions.
